# Differences in vulnerability to desiccating stress between corneal and conjunctival epithelium in rabbit models of short-term ocular surface exposure

**DOI:** 10.1038/s41598-022-21478-9

**Published:** 2022-10-08

**Authors:** Hyun Sun Jeon, Boram Kang, Xuemin Li, Jong Suk Song

**Affiliations:** 1 Department of Ophthalmology, Korea University College of Medicine, Seoul, South Korea; 2 Department of Ophthalmology, Seoul National University College of Medicine, Seoul, South Korea

**Keywords:** Eye diseases, Experimental models of disease

## Abstract

We evaluate the difference in vulnerability to desiccating stress (DS) between the corneal and conjunctival epithelia to understand different ocular surface staining patterns in dry eye patients. We generated a rabbit model of short-term exposure keratopathy. To induce DS in the ocular surface, rabbit right eyelids were opened for 30 min, with blinking once/minute. Corneal staining scores increased from 3-min post-DS exposure, while conjunctival staining increased from 20-min post-DS. At 20 min, the tear MUC5AC level doubled as compared to pre-DS (*p* = 0.007). In Western blot analysis, conjunctival AQP5, MUC5AC, and CFTR expression increased significantly in response to DS, compared to control (*p* = 0.039, 0.002, 0.039, respectively). Immunohistochemistry for CD31 and LYVE-1 were performed. CD31-positive cells and lymphatic space surrounded by LYVE-1-positive cells increased significantly in conjunctival tissue post-DS, compared to control (*p* = 0.0006, *p* < 0.0001, respectively). Surface damage was worse in the corneal than in the conjunctival epithelium after DS, by scanning electron microscopy. This study showed that the cornea and conjunctival epithelium show differences in vulnerability to DS. Increased blood vessels and dilated lymphatics, accompanied by increased conjunctival epithelial AQP5, MUC5AC, and CFTR expression, underlie the protective mechanism of the conjunctiva to desiccating stress.

## Introduction

Ocular surface staining with fluorescein is extensively used to highlight corneal and conjunctival damage in the diagnosis of dry eye disease (DED)^1^. The presence of ocular surface staining represents corneal epithelial barrier disruption and/or conjunctival epithelial damage^2^. Moreover, the nature and pattern of punctate ocular surface staining in DED provides useful clinical information^3^. Various ocular surface scales have been developed to assess ocular surface states. These employ different methods to score ocular surface staining: some include the conjunctiva, while others include only the cornea^4–7^. Although epitheliopathy is commonly observed in DED and contributes to tear film instability^8^, some DED patients demonstrate no ocular surface staining, and only corneal staining without conjunctival staining, or vice versa, which implies that the staining patterns of the cornea and conjunctiva derive from different mechanisms^9,10^. A study by Yang et al.^11^ reported that conjunctival staining scores may be useful to measure ocular surface inflammation in DED, while corneal staining scores did not show any relationship with mRNA levels of various inflammatory cytokines. 

Over the past two decades, growing evidence has shown that inflammation affects the ocular surface in DED^12^. Several animal models have been developed to obtain a deeper understanding of the DED inflammation process^12,13^. Other models of ocular surface disease have been developed that do not necessarily involve ocular surface inflammation or desiccation but instead resort to more subtle changes, like hyperosmolar stress^14,15^. These are categorized into the aqueous-deficient and the evaporative types induced by mechanical or surgical approaches, iatrogenic immune responses, topical eye drops, or blockage of neural pathways^16–18^. A study by Lai et al. in 2015^19^ first reported ocular surface changes in a rabbit model of exposure keratopathy. Whereas the previous studies focused on confirming ocular surface inflammation in DED models, in the present study, to gain understanding of the differences in staining patterns of cornea and conjunctiva in DED, we generated a rabbit model of short-term exposure keratopathy and focused on the ocular surface compensatory mechanism against desiccating stress. We aimed to investigate the difference in vulnerability to desiccating stress between the cornea and conjunctival epithelium. This allowed us to investigate the protective effect of the ocular surface, excluding for the protective effect of the eyelids, against desiccating stress.

## Results

**Figure 1:**
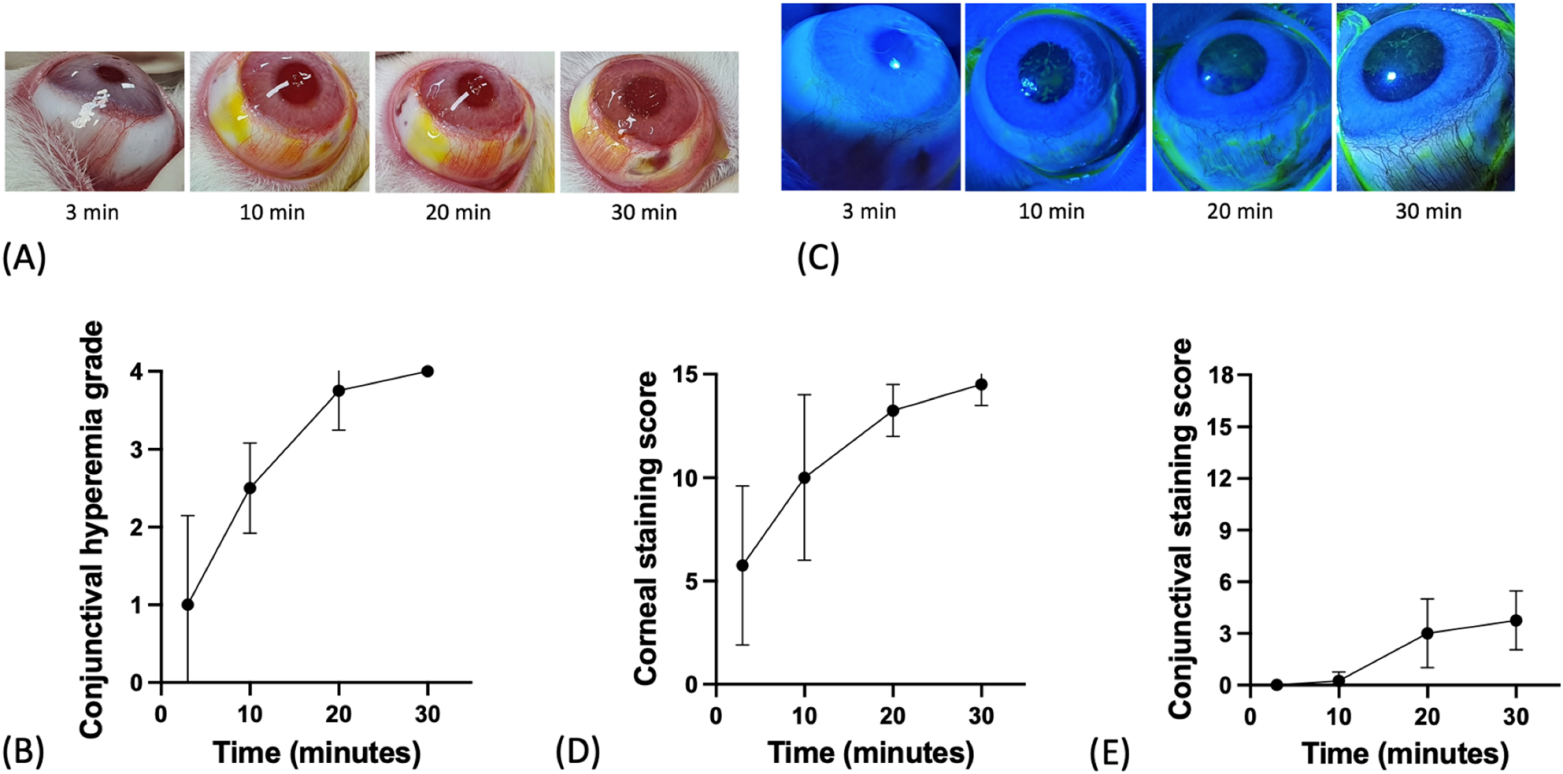
Time-dependent changes in the cornea and conjunctiva of rabbit eyes after exposure. (**A**) Conjunctival hyperemia. (**B**) Changes in conjunctival hyperemia grade. (**C**) Ocular surface fluorescein-staining with cobalt blue filter. (**D**) Changes in corneal staining score. (**E**) Changes in conjunctival staining score. Corneal staining scores continued to increase 3 min after ocular surface exposure, while conjunctival staining started to increase 20 min after exposure.

During the 30-min exposure, rabbit eyes were evaluated at each time point: 3 min, 10 min, 20 min, and 30 min. Conjunctival hyperemia continued to increase after 3 min of exposure and reached the highest score after 30 min (*p* = 0.0023, Friedman test, Fig. 1A,B). Corneal staining scores also continued to increase after 3 min of exposure and reached the highest score after 30 min (*p* = 0.0003, Friedman test, Fig. 1C,D), while conjunctival staining scores remained normal up to 10 min of exposure, started to increase after 20 min of exposure, and remained at a constant level up to 30 min of exposure (*p* = 0.0002, Friedman test, Fig. 1E).

**Figure 2:**
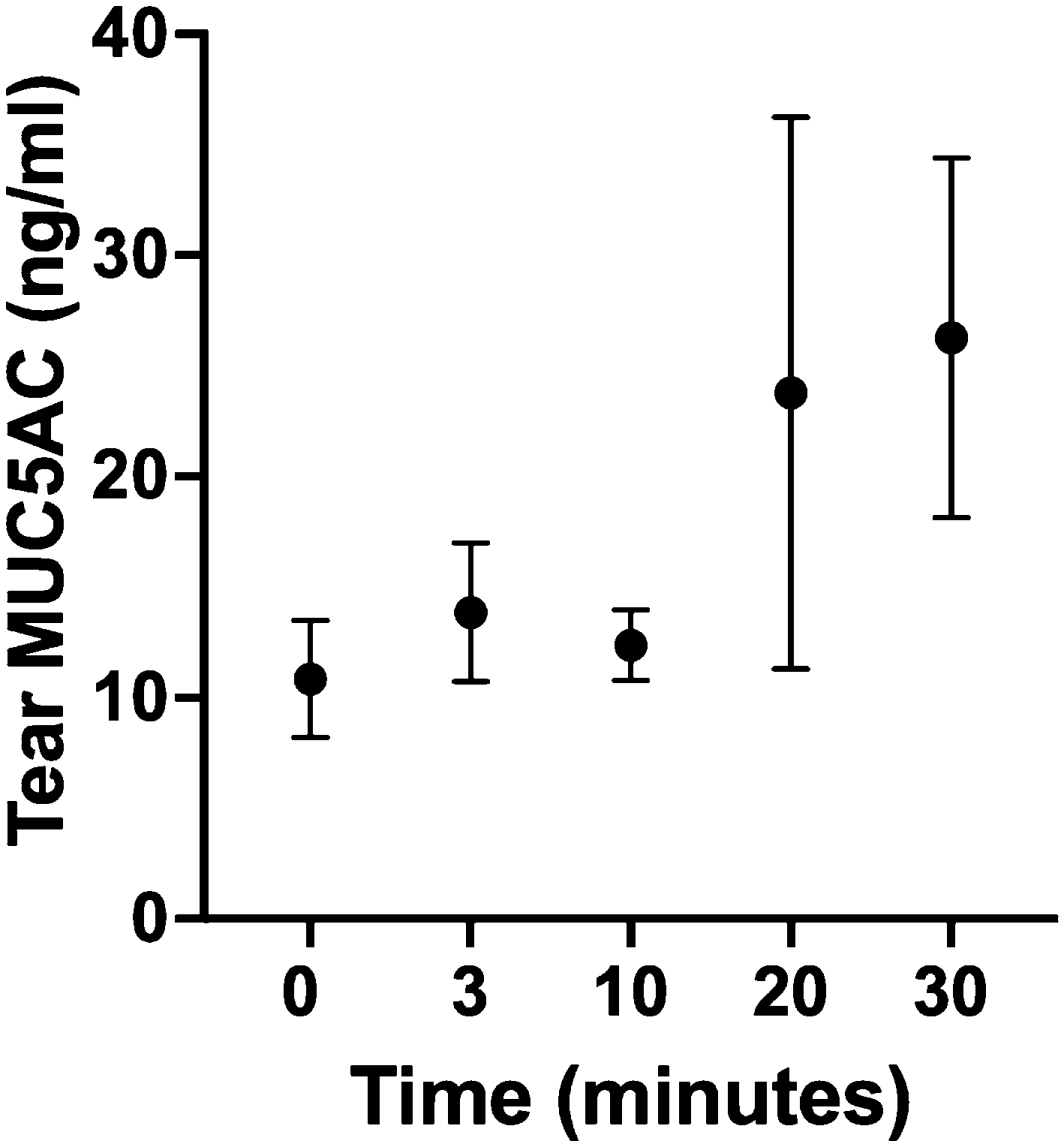
Changes in MUC5AC concentrations in rabbit tear samples after continuous eyelid opening. After 20 min of exposure, the tear MUC5AC level was doubled as compared to that at baseline (*p* = 0.007, Friedman test).

After 20 min of exposure, the mean tear MUC5AC concentration, which was assessed using an enzyme-linked immunosorbent assay had doubled as compared to the baseline and significantly increased (*p* = 0.007, Friedman test, Fig. 2).

**Figure 3:**
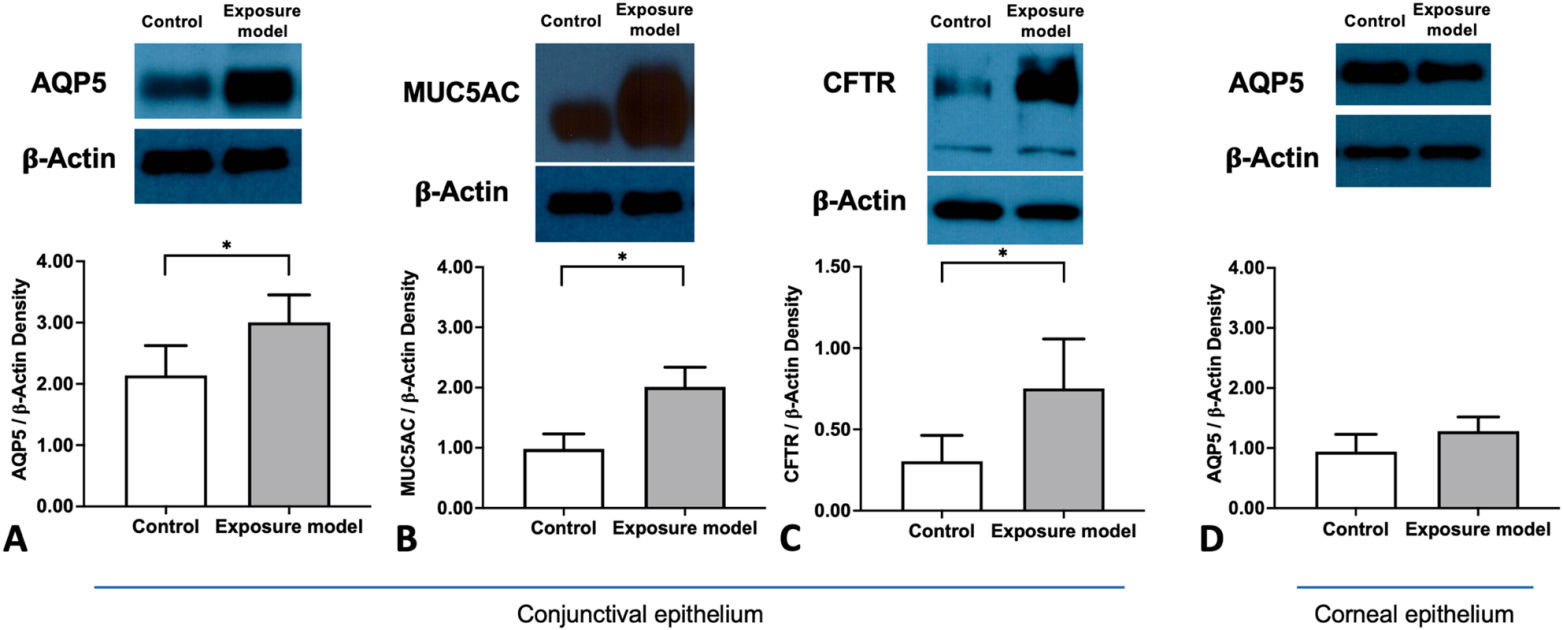
Comparison of the expression of AQP5, MUC5AC, and CFTR in the conjunctiva (A‒C) and cornea (D) between the control and exposure models. (**A**) Expression of AQP5. (**B**) Expression of MUC5AC. (**C**) Expression of CFTR. (**D**) Expression of AQP5 in the cornea. Original blots of Fig. 3A-3D are presented in Supplementary Figure S1-S4.

We measured protein levels of AQP5 in corneal impression cytology samples, and those of AQP5, MUC5AC and CFTR were measured in conjunctival impression cytology samples (Fig. 3). In comparison to control, the expression of AQP5 (*p* = 0.039, Mann–Whitney U test, Fig. 3A), MUC5AC (*p* = 0.002, Mann–Whitney U test, Fig. 3B), and CFTR (*p* = 0.039, Mann–Whitney U test, Fig. 3C) in conjunctiva significantly increased after 30 min of exposure. However, the expression of AQP5 in cornea had no significant changes (*p* = 1.118, Mann–Whitney U test, Fig. 3D).

**Figure 4:**
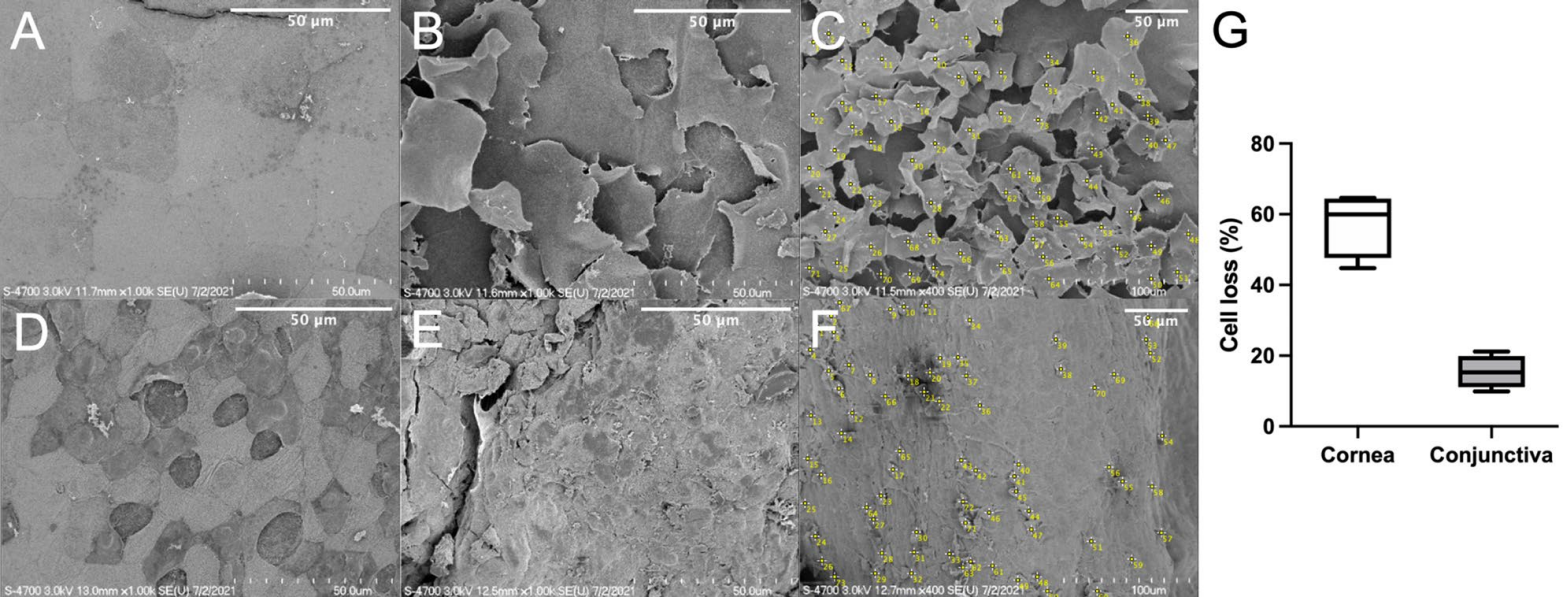
Comparison of changes in the corneal (**A**–**C**) and conjunctival (**D**–**F**) epithelium as observed by scanning electron microscopy in the control (**A**, **D**) and exposure rabbit model 30 min after exposure (**B**, **C**, **E**, **F**). Percentage of cell loss, which shows epithelial cell sloughing or morphologic change was significantly higher in cornea than conjunctiva (**C**, **F**, **G**). Bar: 50 μm.

Scanning electron microscopy examination of the control group on corneal surface displayed no superficial epithelial sloughing or intercellular gaps (Fig. 4A) and that of on conjunctival surface displayed typical irregular polygonal epithelial cells and randomly interspersed goblet cell apertures between epithelial cells (Fig. 4D). After 30 min of exposure, more sloughing of epithelial cells and morphology changes were observed in the cornea (Fig. 4B,C) than in the conjunctiva (Fig. 4E, F). Mean percentage of cell loss, which shows epithelial cell sloughing or morphologic change was significantly higher in cornea (57.3 ± 9.2%) than conjunctiva (15.4 ± 4.6%; *p* = 0.028, Mann–Whitney test, Fig. 4G).

**Figure 5:**
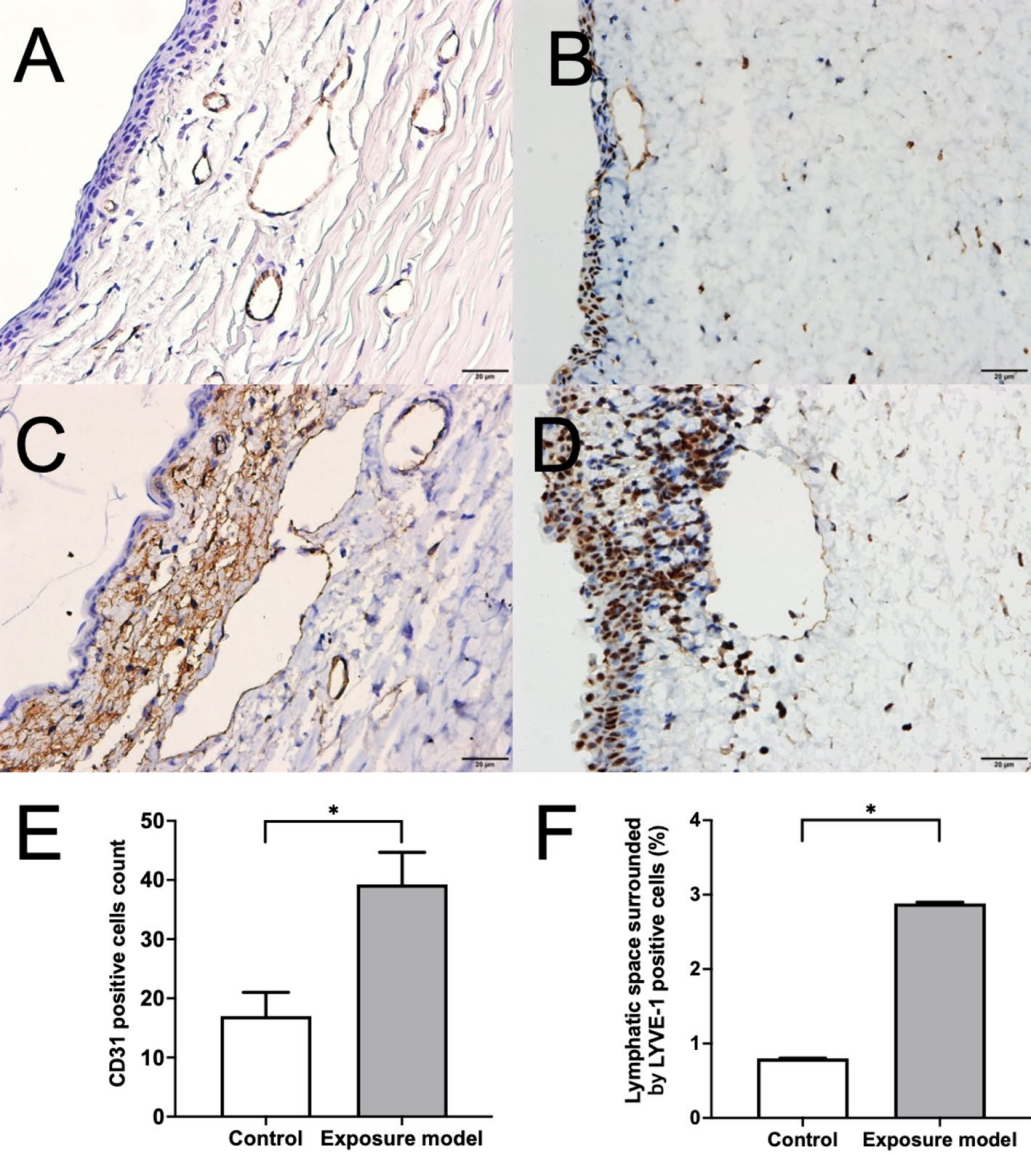
Immunohistochemistry of CD31 (**A**, **C**) and LYVE-1 (**B**, **D**) in serial sections of the conjunctiva of control (**A**, **B**) and exposure rabbit model (**C**, **D**). Comparisons of the number of CD31-positive cells (**E**) and the lymphatic space surrounded by LYVE-1-positive cells (**F**) between control and exposure model rabbits. Number of CD31-positive cells and the lymphatic space surrounded by LYVE-1-positive cells were significantly increased in conjunctival tissue after ocular surface exposure as compared with the control (*p* = 0.0006, *p* < 0.0001, Mann–Whitney U test, respectively).

To understand the compensatory mechanisms of lymphatic and blood vessels involved in early response after desiccating stress, the tissue sections were used for immunohistochemistry of CD31 (as vascular endothelial marker) and lymphatic vessel endothelial hyaluronan (HA) receptor 1 (LYVE-1; as lymphatic endothelial marker). The number of CD31-positive cells was significantly increased after ocular surface exposure in conjunctival tissue, as compared with control tissue (*p* = 0.0006, Mann–Whitney U test; Fig. 5A,C,E). The lymphatic space surrounded by LYVE-1-positive cells was significantly increased after ocular surface exposure in conjunctival tissue, as compared with control tissue (*p* < 0.0001, Mann–Whitney U test; Fig. 5B,D,F).

## Discussion

The present study demonstrated differences in the vulnerability of the cornea and conjunctiva to desiccating stress in a rabbit model of short-term exposure keratopathy. Specifically, our data showed that increased expression of AQP5, MUC5AC, and CFTR, accompanied by enlarged blood and lymphatic vessels in the conjunctiva, may play a crucial role in protecting the conjunctiva from desiccating stress.

Mucin plays important roles in the ocular surface and can be classified broadly according to its structural characteristics as secreted mucins and membrane-associated mucins^20^. Of the secreted mucins, gel-forming mucin MUC5AC is believed to be expressed most prevalently on the ocular surface and is secreted into the tear fluid by the conjunctival goblet cells^20–22^. Previously, the tear MUC5AC concentration was reported to be correlated significantly with goblet cell density^10^ and the severity of ocular surface staining^23^. Thus, it was suggested that tear MUC5AC is a disease-relevant biomarker for conjunctival goblet cells^23^. Several studies have reported decreased MUC5AC secretion in tear fluid and conjunctival epithelial cells in patients with DED^20,21,24,25^. However, in our model of short-term exposure keratopathy, tear MUC5AC levels were significantly increased after 20 min of exposure, indicating that a short-term compensatory mechanism for desiccating stress was triggered. The expression of MUC5AC, as determined by western blot analysis, also confirmed increased expression of MUC5AC in the exposure model eyes as compared to control eyes. Previously, Bhattacharya et al. reported that conjunctival epithelium AQP5 and MUC5AC were expressed in synchrony in response to acute surgically induced dry eye stress, as a compensatory mechanism to restore ocular surface homeostasis^26^. It has been shown that dry eye-related inflammatory mediators (TNFα, IL-1β, IL6/IL17, and prostaglandin E2) upregulate *MUC5AC* mRNA expression^27^. This should be distinguished from the chronic disease condition, in which the goblet cell number and MUC5AC concentration are decreased, indicating that the compensatory mechanism is compromised^26^.

Aquaporins (AQPs) are a group of transmembrane water-channel proteins that mediate the passage of water molecules^28,29^. AQP5 is typically expressed on the apical membrane of the acinar and ductal epithelial cells in human lacrimal glands^30^. AQP3 and AQP5 have also been identified in conjunctival epithelium^31,31^. In 2015, Bhattacharya et al. reported the pivotal role of the conjunctiva in the maintenance of ocular surface homeostasis in rabbits with bilateral resection of the main lacrimal gland and reported that AQP4 and AQP5 are possibly involved in restoring the ocular surface fluids^33^. The expression of AQP5 and MUC5AC has been either positively or negatively correlated in the respiratory track epithelium in different reports^34–36^. Similarly, several studies have reported that close regulatory mechanisms exists between AQP5 and MUC5AC at the ocular surface. In the present study, expression of both AQP5 and MUC5AC were significantly increased in the exposure model as compared to in the control eyes, which supported the findings of previous studies.

Cystic fibrosis transmembrane conductance regulator (CFTR) is a cAMP-regulated chloride channel expressed in various secretory epithelia, including airways, gastrointestinal organs, salivary glands, sweat glands, and the ocular surface. When defective, it may cause cystic fibrosis, the most common genetic disease among Caucasians^37,38^. Several studies have reported DED in patients with cystic fibrosis, suggesting the potential influence of CFTR in tear secretion^29,37,39–43^. Recently, Berczeli et al. reported decreased tear secretion and impaired ocular surface integrity in *CFTR*-knockout mice^38^. In our study, the expression of CFTR in the conjunctival epithelium was significantly increased in eyes exposed to short-term desiccating stress as compared to control eyes, which implies transient increase in fluid efflux as a compensatory mechanism of the conjunctiva to desiccating stress.

Lymphatics play an important role in generating immunoinflammatory responses in peripheral tissues by directing antigen-presenting cells from the periphery to the draining lymph nodes^44,45^. Recently, there has been growing recognition of the importance of corneal lymphatic vessels in mediating the activation and movement of immune cells at the corneal surface in DED^46,47^. This recognition has grown significantly with the development of reliable lymphatic markers. Lymphatic vessel endothelial hyaluronan (HA) receptor 1 (LYVE-1) is a member of the Link superfamily of HA-binding proteins that is highly expressed on lymphatic vessels and serves as a lymphatic-specific marker^48–50^. In the present study, the lymphatic space surrounded by LYVE-1-positive cells was significantly increased in conjunctival tissue of eyes after ocular surface exposure as compared to in the control tissues. The number of CD31-positive cells was also increased after short term desiccating stress. CD31 is an endothelial cell surface marker, indicating blood vessels, whereas LYVE-1 functions in cell adhesion/transmigration in lymphangiogenesis^46^. These results indicate that dilatation of lymphatic and blood vessels could be an early response after desiccating stress and suggest that lymphangiogenesis along with hemangiogenesis could form part of a compensatory mechanism to maintain homeostasis of the ocular surface.

There are several limitations in this study. First, this study used a small number of animals to draw conclusions. However, the results were consistent in showing the difference between the cornea and conjunctiva and revealing the protective effect of the conjunctiva. Second, the use of normal saline in the process of tear sample collection could have affected the ocular surface of rabbit models by a temporary wetting effect. To minimize this effect and obtain consistent results, we standardized the experimental conditions by using a set sequence of examinations. Third, we only evaluated up to a 30-min exposure, which does not reflect the effects of long-term exposure of the ocular surface. However, since our main purpose was to confirm the protective effect of the conjunctiva or cornea against desiccating stress, we consider that this was sufficient time to confirm a short-term protective effect. Last, we could not evaluate the recovery of the ocular surface over time after short-term exposure.

In conclusion, in this study, we demonstrated a difference in vulnerability to desiccating stress between the corneal and conjunctival epithelium and confirmed that this vulnerability difference causes the difference in corneal and conjunctival staining in dry eye patients. The increase in blood vessels and the dilatation of lymphatic vessels in the conjunctival tissue, accompanied by increased expression of AQP5, MUC5AC, and CFTR in the conjunctival epithelium, seem to underlie the protective mechanism of the conjunctiva to desiccating stress. Further studies with models such as Sjögren’s syndrome and graft versus host disease, in which the compensatory mechanism of the conjunctiva is damaged, will be helpful to better understand the compensatory mechanism of conjunctiva.

## Methods

### Rabbit model of short-term exposure keratopathy

New Zealand white rabbits (weight, 2.0‒2.2 kg) were used in this study. The study was carried out in compliance with the ARRIVE guidelines. All procedures adhered to the Association for Research in Vision and Ophthalmology (ARVO) Statement for the Use of Animals in Ophthalmic and Vision Research (ARVO Animal Policy). Approval for this study was obtained from the Korea University Guro Hospital Institutional Review Board, Seoul, South Korea (KOREA-2021–0122).

All in vivo experimental procedures (including ocular surface staining, tear sample collection, and impression cytology) were performed under general anesthesia induced by the intramuscular injection of ketamine hydrochloride (35 mg/kg) and xylazine hydrochloride (5 mg/kg)^50^. The right eyes of the rabbits were used for all experiments, and the left eyes were left untreated and were used as controls. The interpalpebral fissures were kept wide-open for 30 min to ensure the exposure of the central cornea, limbus, and perilimbal conjunctiva, with blinking performed manually once every minute. Eyes were evaluated at each time point: 3 min, 10 min, 20 min, and 30 min.

### Ocular surface staining and conjunctival hyperemia

The cornea and conjunctiva of rabbits were examined under a portable slit-lamp microscope with a cobalt blue filter, under general anesthesia, after instillation of 1 drop 1% fluorescein solution. A yellow filter was used to evaluate the staining of the conjunctiva in detail. For corneal fluorescein staining (CFS) scores, fluorescein sodium-impregnated paper strips (Haag-Streit, Bern, Switzerland) were applied to the upper bulbar surface after retracting the upper eyelid and after wetting the end of the strip with 5 μL saline.

The ocular staining score was assessed by a single experienced ophthalmologist according to the standard National Eye Institute (NEI) grading system. Briefly, the cornea was divided into five areas (central, superior, nasal, inferior, and temporal); punctate fluorescein staining in each area was graded on a scale of 0 to 3, and the total score (0–15) is the sum of scores for all five areas. The conjunctiva was divided into six areas and the total score (0–18) is the sum of scores for all six areas^4^. The conjunctival hyperemia was graded using the Efron scale for conjunctival redness, which consists of five grades (0–4)^51^.

### Tear sample collection and enzyme-linked immunosorbent assay

Tear samples were collected from the rabbit eyes baseline and after 3, 10, 20, and 30 min of exposure of the conjunctiva and cornea. Sixty microliters of normal saline was gently applied to the inferior fornix using a micropipette, twice. Thereafter, a total volume of 120 μl of a tear sample was gently collected^52^. Tear samples were used to measure MUC5AC levels, to investigate conjunctival goblet cell mucin secretion into the tears. The level of MUC5AC in the tear sample was assessed using an enzyme-linked immunosorbent kit for rabbit MUC5AC (MyBiosource, San Diego, CA, USA). All measurements were conducted according to the manufacturer’s protocol, using a microplate spectrophotometer (Spectramax Plus® 384; Molecular Devices, Sunnyvale, CA, USA).

### Impression cytology and western blot analysis

After the last tear sample was collected after 30 min of eye exposure, corneal and conjunctival impression cytology was performed. After instilling an 0.5% proparacaine hydrochloride eye drop (Alcaine ®, Bausch & Lomb, Rochester, NY, USA). An 8-mm-diameter nitrocellulose membrane (Millipore, Bedford, MA, USA) was applied to the cornea and conjunctiva. The membrane was then gently peeled off with smooth forceps. The membrane was immediately immersed into a well that was filled with fixing solution. Hematoxylin and eosin (H&E) and periodic acid‒Schiff (PAS) staining were used for histological staining of the membrane in the specimen^53^.

Tissue cell extracts from corneal impression cytology were subjected to western blot analysis to measure protein levels of AQP5. Those from conjunctival impression cytology were subjected to western blot analysis to measure protein levels of AQP5, MUC5AC, and CFTR. Primary antibodies against AQP5 (1:1000; ab92320, Abcam, Cambridge, UK), MUC5AC (1:1000; ab198294, Abcam, Cambridge, UK), CFTR (1:1000; MAB3482, Sigma-Aldrich, Missouri, USA), and β-actin (1:10,000, No. 5125; Cell Signaling Technology, Danvers, MA, USA) were used. The impression cytology from left eyes, which left untreated were used as controls. The original images as they were saved during the experiment are presented in Figure S1–S4 as supplementary files. We only stored the protein band images of interest and images of full-length blots were not presented.

### Scanning electron microscopy

After exposure for 30 min, for scanning electron microscopy (SEM) and immunohistochemistry, rabbits were euthanized using a CO2 chamber under general anesthesia. The corneal and conjunctival tissues were gently excised after sacrifice and were prefixed in 2% glutaraldehyde in 0.1 M phosphate buffer. Samples were then post-fixed for 2 h in 1% osmic acid dissolved in phosphate-buffered saline for SEM. Then, the corneal and conjunctival tissues were treated in a graded series of ethanol and t-butyl alcohol, dried in a freeze dryer (ES-2030; Hitachi, Tokyo, Japan), and coated with platinum using an ion coater (IB-5; Eiko, Ibaraki, Japan). The appearance of the corneal and conjunctival epithelial surface was observed via field emission-SEM (S-4700; Hitachi)^54^. We defined the percentage of cell loss as the ratio of the cell loss, which shows epithelial cell sloughing or morphologic change to the total epithelial cells in each four representative digital images from SEM to quantify the epithelial cell loss. First, total area of each representative images was calculated using ‘set scale’ based on the known distance from SEM using ImageJ (http://imagej.nih.gov/ij/; provided in the public domain by the National Institutes of Health, Bethesda, MD, USA). Second, area of each corneal and conjunctival epithelial cell was calculated from ‘polygon selection tool’. Third, count of total epithelial cells in each representative image was calculated as the total area of each representative images divided by the area of each epithelial cell. Fourth, the number of cell loss, which shows epithelial cell sloughing or morphologic change was calculated using ‘multi-point tool’ (Fig. 4C,F). Finally, the percentage of cell loss was calculated as ratio of the cell loss to the total epithelial cells in the representative images.

### Immunohistochemistry

The anterior segment of each eyeball was surgically removed and fixed in 10% neutral buffered formalin and was then embedded in paraffin. Paraffin-embedded tissues were cut into 4-μm sections with a microtome (Leica RM 2255; Leica, Bannockburn, IL, USA), and tissue sections were placed on microscope slides. After deparaffinization of the tissue sections with xylene, tissue sections were immersed in a graded series of ethanol and phosphate-buffered saine. Serial sections were used for immunohistochemistry of CD31 (as vascular endothelial marker) and LYVE-1 (as lymphatic endothelial marker). Primary antibodies were commercially obtained for CD31 (1:500; Santa Cruz Biotechnology, Santa Cruz, California), and LYVE-1 (1:100; Abcam Inc, Cambridge, Massachusetts). A rabbit-specific HRP/DAB (ABC) Detection IHC Kit (ab64261; Abcam) was used for secondary antibody-based detection according to the manufacturer’s instructions. The tissue sections were observed under light microscopy at 400 × magnification, and digital images were taken with an Olympus BX51 microscope and a DP72 camera (Olympus Optical Co., Ltd., Tokyo, Japan). The cells positive for CD31 were determined by analyzing four visual fields on conjunctival biopsy and results are reported as cells per square millimeter. The lymphatic space surrounded by LYVE-1-positive cells (%) were defined as percentage of the summed area surrounded by LYVE-1 positive cells per total area observed. The mean CD31-positive cell count and the lymphatic space surrounded by LYVE-1-positive cells (%) were compared between the exposure model eye and the control eye.

### Statistical analyses

Statistical analyses were performed using the Mann‒Whitney U test and Wilcoxon signed-rank test in SPSS version 20.0 (IBM SPSS, Inc., Chicago, IL, USA). Values are expressed as the median and interquartile range. *P* < 0.05 was considered statistically significant.

## Supplementary Information

Supplementary Information.

## Data Availability

The datasets generated during and/or analysed during the current study are available from the corresponding author on reasonable request.

## References

[1] Wolffsohn JS, Arita R, Chalmers R, Djalilian A, Dogru M, Dumbleton K (2017). TFOS DEWS II diagnostic methodology report. Ocul. Surf..

[2] Craig JP, Nelson JD, Azar DT, Belmonte C, Bron AJ, Chauhan SK (2017). TFOS DEWS II report executive summary. Ocul. Surf..

[3] Bron AJ, Argüeso P, Irkec M, Bright FV (2015). Clinical staining of the ocular surface: Mechanisms and interpretations. Prog. Retin. Eye Res..

[4] Lemp MA (1995). Report of the national eye institute/industry workshop on clinical trials in dry eyes. CLAO J..

[5] Whitcher JP, Shiboski CH, Shiboski SC, Heidenreich AM, Kitagawa K, Zhang S (2010). A simplified quantitative method for assessing keratoconjunctivitis sicca from the sjogren’s syndrome international registry. Am. J. Ophthalmol..

[6] Bron AJ, Evans VE, Smith JA (2003). Grading of corneal and conjunctival staining in the context of other dry eye tests. Cornea.

[7] Doughty MJ (2013). Rose bengal staining as an assessment of ocular surface damage and recovery in dry eye disease-a review. Cont. Lens Anterior Eye..

[8] Tsubota K, Pflugfelder SC, Liu Z, Baudouin C, Kim HM, Messmer EM (2020). Defining dry eye from a clinical perspective. Int J Mol Sci.

[9] Pflugfelder SC, De Paiva CS, Villarreal AL, Stern ME (2008). Effects of sequential artificial tear and cyclosporine emulsion therapy on conjunctival goblet cell density and transforming growth factor-beta2 production. Cornea.

[10] Uchino Y, Uchino M, Yokoi N, Dogru M, Kawashima M, Komuro A (2016). Impact of cigarette smoking on tear function and correlation between conjunctival goblet cells and tear MUC5AC concentration in office workers. Sci Rep..

[11] Yang S, Lee HJ, Kim DY, Shin S, Barabino S, Chung SH (2019). The use of conjunctival staining to measure ocular surface inflammation in patients with dry eye. Cornea.

[12] Baudouin C, Irkec M, Messmer EM, Benitez-Del-Castillo JM, Bonini S, Figueiredo FC (2017). Clinical impact of inflammation in dry eye disease. proceedings of the ODISSEY group meeting. Acta. Ophthalmol..

[13] Chang YA, Wu YY, Lin CT, Kawasumi M, Wu CH, Kao SY (2021). Animal models of dry eye: Their strengths and limitations for studying human dry eye disease. J. Chin. Med. Assoc..

[14] Luo L, Li DQ, Corrales RM, Pflugfelder SC (2005). Hyperosmolar saline is a proinflammatory stress on. He mouse ocular surface. Eye Contact Lens.

[15] Guzman M, Miglio M, Keitelman I, Shiromizu CM, Sabbione F, Fuentes F (2020). Transient tear hyperosmolarity disrupts the neuroimmune homeostasis of the ocular surface and facilitates dry eye onset. Immunology.

[16] Dursun D, Wang M, Monroy D, Li DQ, Lokeshwar BL, Stern ME (2002). A mouse model of keratoconjunctivitis sicca. Invest. Ophthalmol. Vis. Sci..

[17] El Annan J, Chauhan SK, Ecoiffier T, Zhang Q, Saban DR, Dana R (2009). Characterization of effector T cells in dry eye disease. Invest. Ophthalmol. Vis. Sci..

[18] Chen Y, Chauhan SK, Lee HS, Stevenson W, Schaumburg CS, Sadrai Z (2013). Effect of desiccating environmental stress versus systemic muscarinic AChR blockade on dry eye immunopathogenesis. Invest. Ophthalmol. Vis. Sci..

[19] Lai CT, Yao WC, Lin SY, Liu HY, Chang HW, Hu FR (2015). Changes of ocular surface and the inflammatory response in a rabbit model of short-term exposure keratopathy. PLoS ONE.

[20] Zhao H, Jumblatt JE, Wood TO, Jumblatt MM (2001). Quantification of MUC5AC protein in human tears. Cornea.

[21] Gipson IK, Argüeso P (2003). Role of mucins in the function of the corneal and conjunctival epithelia. Int. Rev. Cytol..

[22] Hori Y (2018). Secreted mucins on the ocular surface. Invest. Ophthalmol. Vis. Sci.

[23] Khimani KS, Go JA, De Souza RG, Mitchell T, Yu Z, de Paiva CS (2020). Regional comparison of goblet cell number and area in exposed and covered dry eyes and their correlation with tear MUC5AC. Sci. Rep..

[24] Argüeso P, Balaram M, Spurr-Michaud S, Keutmann HT, Dana MR, Gipson IK (2002). Decreased levels of the goblet cell mucin MUC5AC in tears of patients with Sjögren syndrome. Invest. Ophthalmol. Vis. Sci..

[25] Shimazaki-Den S, Dogru M, Higa K, Shimazaki J (2013). Symptoms, visual function, and mucin expression of eyes with tear film instability. Cornea.

[26] Bhattacharya D, Yu L, Wang M (2017). Expression patterns of conjunctival mucin 5AC and aquaporin 5 in response to acute dry eye stress. PLoS ONE.

[27] Rose MC, Voynow JA (2006). Respiratory tract mucin genes and mucin glycoproteins in health and disease. Physiol. Rev..

[28] Kordowitzki P, Kranc W, Bryl R, Kempisty B, Skowronska A, Skowronski MT (2020). The relevance of aquaporins for the physiology, pathology, and aging of the female reproductive system in mammals. Cells.

[29] Levin MH, Verkman AS (2006). Aquaporins and CFTR in ocular epithelial fluid transport. J. Membr. Biol..

[30] Ishida N, Hirai SI, Mita S (1997). Immunolocalization of aquaporin homologs in mouse lacrimal glands. Biochem. Biophys. Res. Commun..

[31] Levin MH, Verkman AS (2004). Aquaporin-dependent water permeation at the mouse ocular surface: In vivo microfluorimetric measurements in cornea and conjunctiva. Invest. Ophthalmol. Vis. Sci..

[32] Oen H, Cheng P, Turner HC, Alvarez LJ, Candia OA (2006). Identification and localization of aquaporin 5 in the mammalian conjunctival epithelium. Exp. Eye. Res..

[33] Bhattacharya D, Ning Y, Zhao F, Stevenson W, Chen R, Zhang J (2015). Tear production after bilateral main lacrimal gland resection in rabbits. Invest. Ophthalmol. Vis. Sci..

[34] Chen Z, Wang X, Gao L, Bai L, Zhu R, Bai C (2006). Regulation of MUC5AC mucin secretion by depletion of AQP5 in SPC-A1 cells. Biochem. Biophys. Res. Commun..

[35] Zhang ZQ, Zhu ZX, Bai CX, Chen ZH (2011). Aquaporin 5 expression increases mucin production in lung adenocarcinoma. Oncol. Rep..

[36] Wang K, Feng YL, Wen FQ, Chen XR, Ou XM, Xu D (2007). Decreased expression of human aquaporin-5 correlated with mucus overproduction in airways of chronic obstructive pulmonary disease. Acta pharmacol. sin..

[37] Turner HC, Bernstein A, Candia OA (2002). Presence of CFTR in the conjunctival epithelium. Curr. Eye. Res..

[38] Berczeli O, Vizvári E, Katona M, Török D, Szalay L, Rárosi F (2018). Novel insight into the role of CFTR in lacrimal gland duct function in mice. Invest. Ophthalmol. Vis. Sci..

[39] Sheppard JD, Orenstein DM, Chao CC, Butala S, Kowalski RP (1989). The ocular surface in cystic fibrosis. Ophthalmology.

[40] Castagna I, Roszkowska AM, Famà F, Sinicropi S, Ferreri G (2001). The eye in cystic fibrosis. Eur. J. Ophthalmol..

[41] Mrugacz M, Kaczmarski M, Bakunowicz-Lazarczyk A, Zelazowska B, Wysocka J, Minarowska A (2006). IL-8 and IFN-gamma in tear fluid of patients with cystic fibrosis. J. Interferon. Cytokine Res..

[42] Lee HK, Park J, Kim BR, Jun I, Kim TI, Namkung W (2021). Isorhamnetin ameliorates dry eye disease via CFTR activation in mice. Int. J. Mol. Sci..

[43] Pasricha ND, Smith AJ, Levin MH, Schallhorn JM, Verkman AS (2020). Ocular surface potential difference measured in human subjects to study ocular surface ion transport. Transl. Vis. Sci. Technol..

[44] Cursiefen C, Cao J, Chen L, Liu Y, Maruyama K, Jackson D (2004). Inhibition of hemangiogenesis and lymphangiogenesis after normal-risk corneal transplantation by neutralizing VEGF promotes graft survival. Invest. Ophthalmol. Vis. Sci..

[45] Goyal S, Chauhan SK, Dana R (2012). Blockade of prolymphangiogenic vascular endothelial growth factor C in dry eye disease. Arch. Ophthalmol..

[46] Chennakesavalu M, Somala SRR, Dommaraju SR, Peesapati MP, Guo K, Rosenblatt MI (2021). Corneal lymphangiogenesis as a potential target in dry eye disease–A systematic review. Surv. Ophthalmol..

[47] Yang JF, Walia A, Huang YH, Han KY, Rosenblatt MI, Azar DT (2016). Understanding lymphangiogenesis in knockout models, the cornea, and ocular diseases for the development of therapeutic interventions. Surv. Ophthalmol..

[48] Banerji S, Ni J, Wang SX, Clasper S, Su J, Tammi R (1999). LYVE-1, a new homologue of the CD44 glycoprotein, is a lymph-specific receptor for hyaluronan. J. Cell. Biol..

[49] Nakao S, Zandi S, Faez S, Kohno R, Hafezi-Moghadam A (2012). Discontinuous LYVE-1 expression in corneal limbal lymphatics: Dual function as microvalves and immunological hot spots. FASEB J..

[50] Cursiefen C, Schlötzer-Schrehardt U, Küchle M, Sorokin L, Breiteneder-Geleff S, Alitalo K (2002). Lymphatic vessels in vascularized human corneas: Immunohistochemical investigation using LYVE-1 and podoplanin. Invest. Ophthalmol. Vis. Sci..

[51] Efron N (1998). Grading scales for contact lens complications. Ophthalmic. Physiol. Opt..

[52] Eom Y, Song JS, Lee DY, Kim MK, Kang BR, Heo JH (2016). Effect of titanium dioxide anoparticle. Exposure on the ocular surface: An animal study. Ocul Surf..

[53] Singh R, Joseph A, Umapathy T, Tint NL, Dua HS (2005). Impression cytology of the ocular surface. Br. J. Ophthalmol..

[54] Song JS, Heo JH, Kim HM (2012). Protective effects of dispersive viscoelastics on corneal endothelial. Damage in a toxic anterior segment syndrome animal model. Invest. Ophthalmol. Vis. Sci.

