# Supplementary material for: Differences in vulnerability to desiccating stress between corneal and conjunctival epithelium in rabbit models of short-term ocular surface exposure
Source: Sci Rep. 2022 Oct 8;12:16941. doi: 10.1038/s41598-022-21478-9 (PMC9547869; doi:10.1038/s41598-022-21478-9)

**Figure S1.** Uncropped image of Figure 3A, which shows expression of AQP5 in conjunctival epithelium.

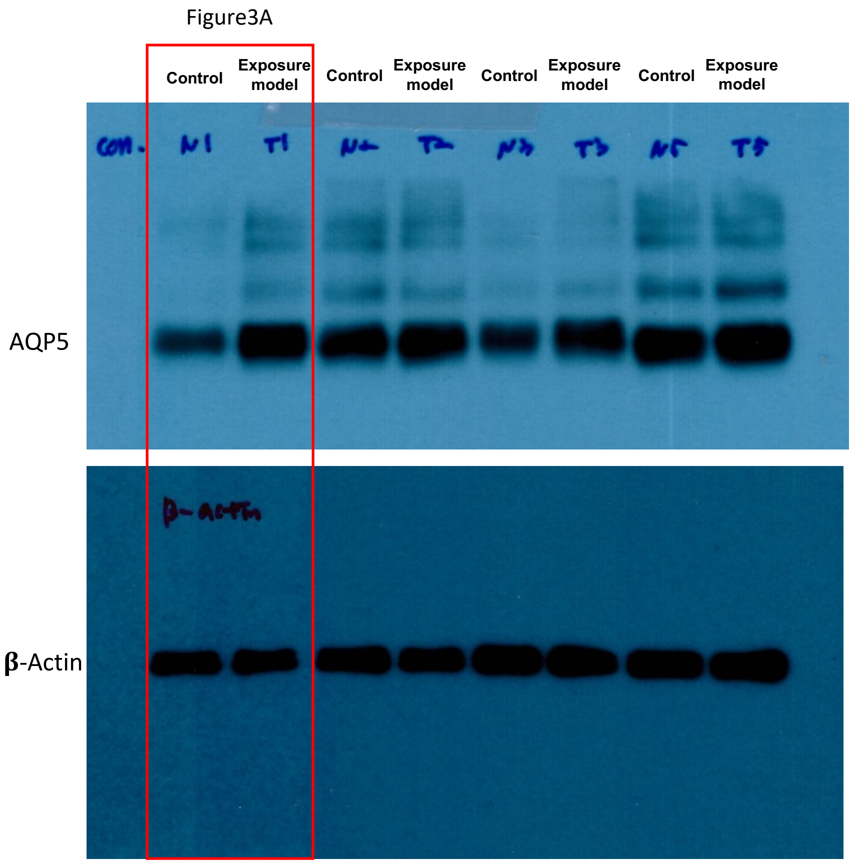

**Figure S2.** Uncropped image of Figure 3B, which shows expression of MUC5AC in conjunctival epithelium.

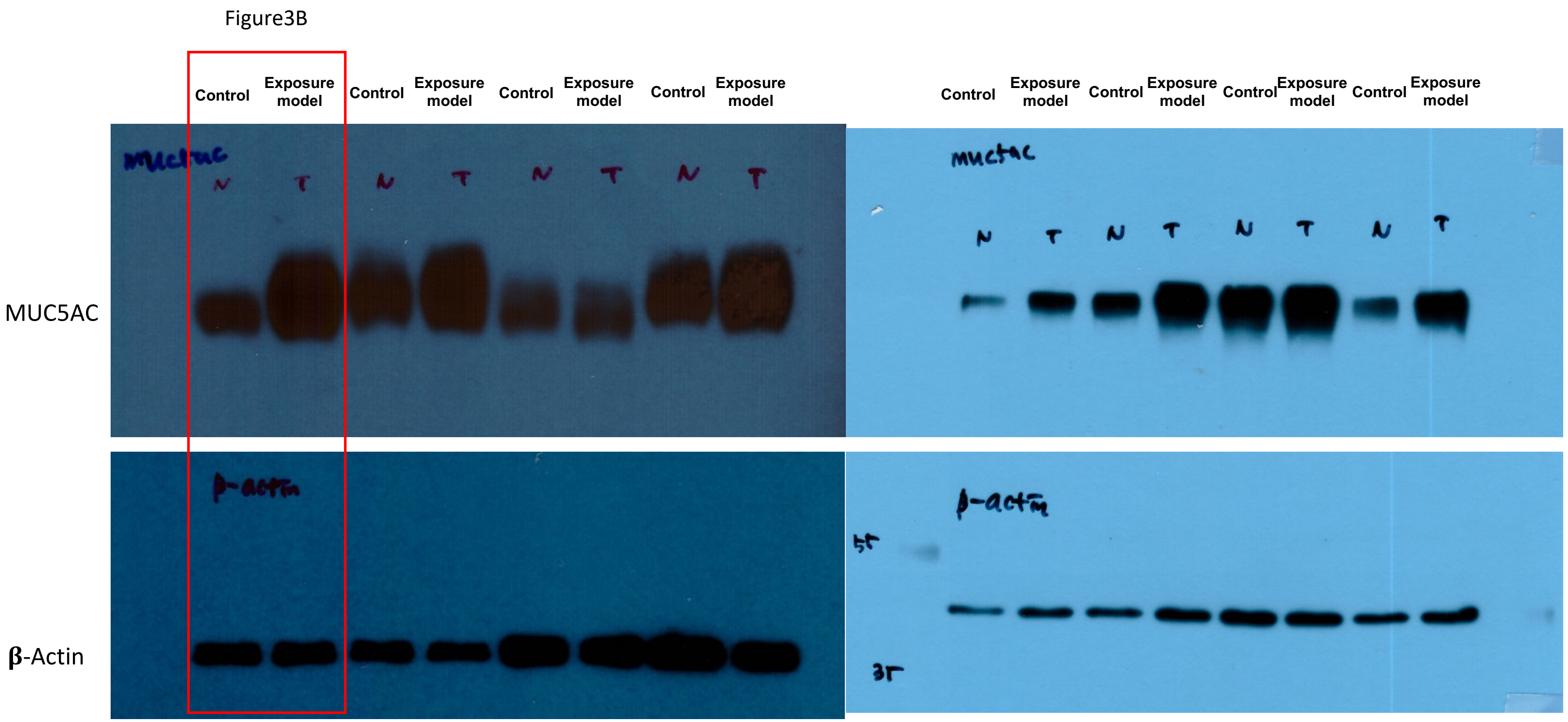

**Figure S3.** Uncropped image of Figure 3C, which shows expression of CFTR in conjunctival epithelium.

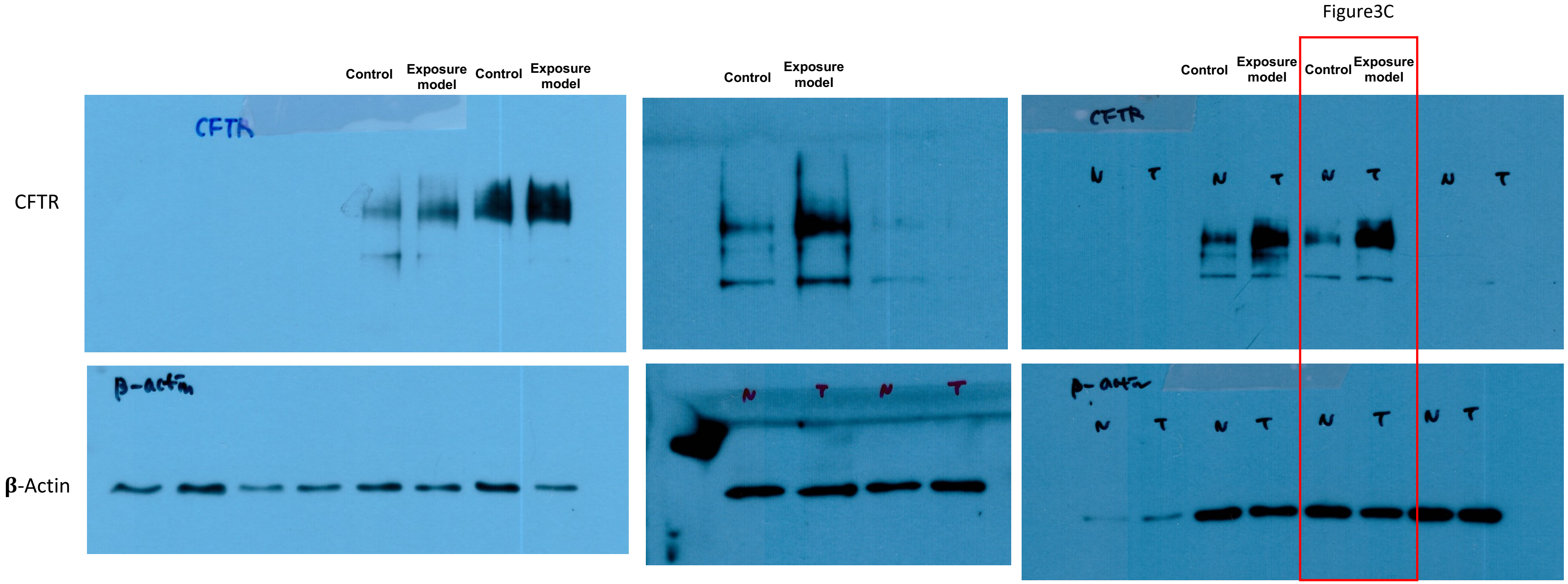

**Figure S4.** Uncropped image of Figure 3D, which shows expression of AQP5 in corneal epithelium.

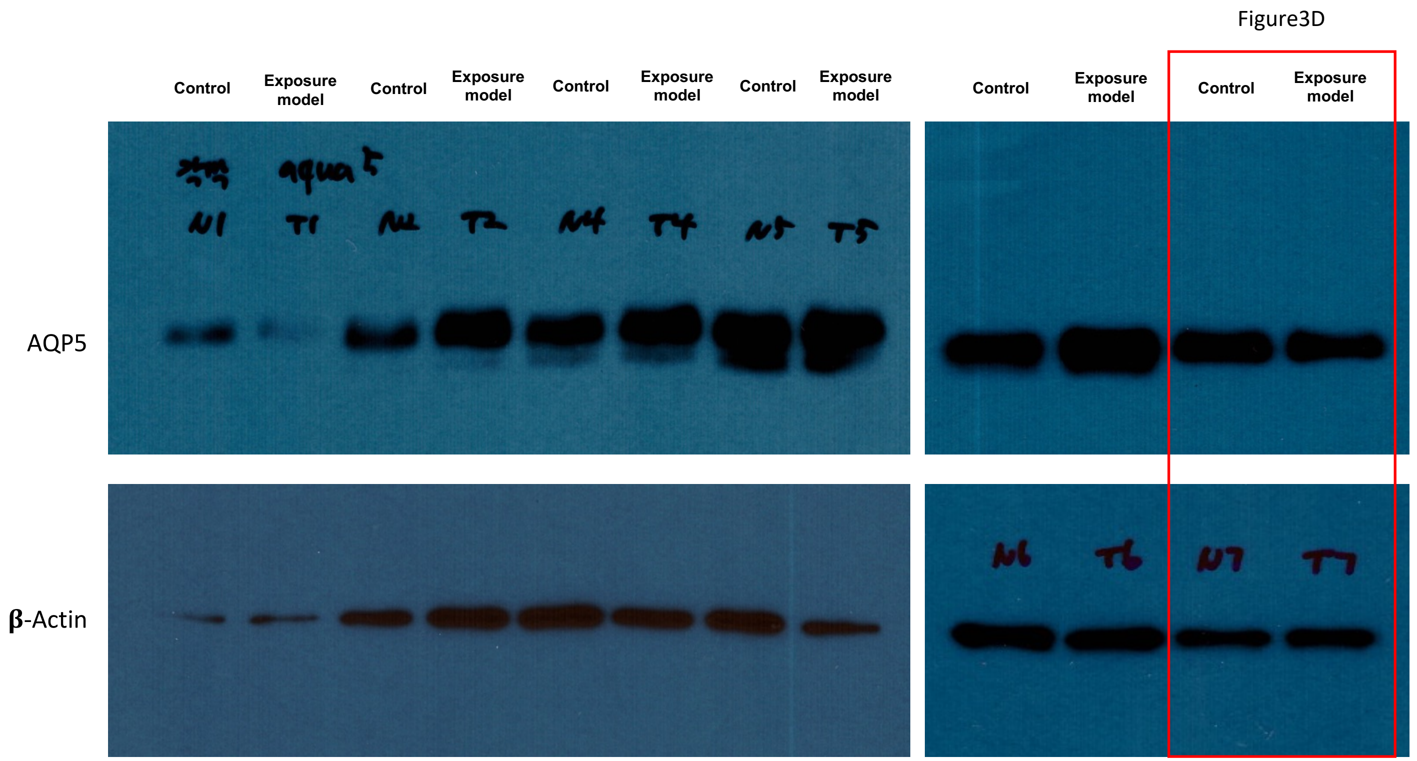

Supplement: Supplementary file 1 — Supplementary Information. [file 41598_2022_21478_MOESM1_ESM.pdf]
